# Sicherheitskultur in der Orthopädie und Unfallchirurgie: Eine qualitative Untersuchung der ärztlichen Perspektive

**DOI:** 10.1007/s00113-020-00917-0

**Published:** 2020-11-10

**Authors:** Isabel Höppchen, Charlotte Ullrich, Michel Wensing, Regina Poß-Doering, Arnold J. Suda

**Affiliations:** 1 Institut für Pflegewissenschaft und -praxis, Paracelsus Medizinische Privatuniversität, Strubergasse 21, 5020 Salzburg, Österreich; 2 Abteilung Allgemeinmedizin und Versorgungsforschung, Universitätsklinikum Heidelberg, Im Neuenheimer Feld 130.3, 69120 Heidelberg, Deutschland; 3 Abteilung für Orthopädie und Traumatologie, AUVA Unfallkrankenhaus Salzburg, Akademisches Lehrkrankenhaus der Paracelsus Medizinischen Privatuniversität, Dr. Franz-Rehrl-Platz 5, 5010 Salzburg, Österreich

**Keywords:** Riskomanagement, Kommunikationskultur, Führung, Ärztliches Team, Unerwünschte Ereignisse, Risk management, Communication culture, Leadership, Medical team, Adverse events

## Abstract

**Hintergrund:**

Krankenhäuser in Deutschland betreiben ein Risikomanagement, welches die Prävention und systematische Aufarbeitung unerwünschter Ereignisse unterstützen kann. Ein wichtiger Aspekt davon ist die Etablierung einer Sicherheitskultur. Die Erhebung der Sicherheitskultur findet im deutschsprachigen Raum bisher selten und fast ausschließlich durch quantitative Instrumente statt. Im Fachbereich Orthopädie und Unfallchirurgie ist in Deutschland eine hohe Zahl an bestätigten Behandlungsfehlern und Risikoaufklärungsmängeln verzeichnet. Deshalb untersucht die vorliegende Studie die Sicherheitskultur in diesem Fachbereich.

**Fragestellung:**

(I) Wie nehmen Ärzte der Orthopädie und Unfallchirurgie den Umgang mit unerwünschten Ereignissen im klinischen Alltag wahr, und (II) was sind die relevanten Bestandteile einer Sicherheitskultur aus ärztlicher Perspektive?

**Material und Methoden:**

Es wurden 14 Einzelinterviews mit Ärzten der Orthopädie und Unfallchirurgie geführt. Die Interviews wurden audioaufgezeichnet, transkribiert und anhand der Thematic Analysis nach Braun und Clarke und des Yorkshire Contributory Factors Framework analysiert. Zur Organisation der Daten wurde die Software MAXQDA verwendet.

**Ergebnisse:**

Es konnte ein starker Einfluss der Führungskräfte auf den Umgang mit unerwünschten Ereignissen im ärztlichen Team festgestellt werden. Von Chefärzten wurde erwartet, eine gute Sicherheitskultur vorbildhaft vorzuleben, da sie durch ihr Verhalten die Handlungsweisen des Teams in sicherheitsrelevanten Situationen beeinflussen.

**Diskussion:**

Der Einbezug von Chefärzten in die Entwicklung von Maßnahmen zur Verbesserung der Sicherheitskultur in der Orthopädie und Unfallchirurgie sollte aufgrund der Bedeutsamkeit hierarchischer Strukturen in Betracht gezogen werden.

**Zusatzmaterial online:**

Die Online-Version dieses Beitrags (10.1007/s00113-020-00917-0) enthält weitere Informationen zu Material und Methoden der Studie. Beitrag und Zusatzmaterial stehen Ihnen auf www.springermedizin.de zur Verfügung. Bitte geben Sie dort den Beitragstitel in die Suche ein, das Zusatzmaterial finden Sie beim Beitrag unter „Ergänzende Inhalte“.

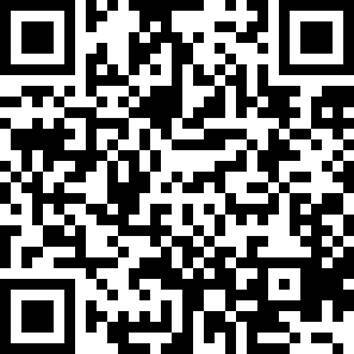

## Abstract (en)

**Background:**

Hospitals in Germany operate risk management, which can support the prevention of adverse events and to systematically process them. Safety culture is one aspect of this. In German-speaking countries, however, surveying the safety culture is still limited and almost exclusively carried out using quantitative instruments. In Germany, the discipline of orthopedics and trauma surgery is characterized by a high number of confirmed treatment errors and lack of risk clarification, Therefore, this study investigated the safety culture in this discipline.

**Objective:**

(I) How do orthopedic and trauma surgeons perceive the handling of adverse events in trauma centers and (II) what are relevant components of the safety culture from a medical perspective?

**Material and methods:**

A qualitative study using 14 semi-structured one-to-one interviews was carried out with physicians in orthopedics and trauma surgery. The interviews were audiotaped, transcribed, and analyzed using the thematic analysis by Braun and Clarke and the Yorkshire contribution factors framework. The MAXQDA software was used to organize data.

**Results:**

A strong influence of senior staff on the handling of adverse events in the medical team could be determined. It was expected that senior staff members have a strong model role function. They were expected to exemplify a high safety culture, because their behavior influences the team’s behavior in patient safety-related situations.

**Conclusion:**

The involvement of senior staff in the development of interventions to improve the safety culture in orthopedics and trauma surgery should be considered due to the importance of hierarchical structures.

## Hintergrund und Zielsetzung

Aufgrund gesetzlicher Vorgaben zur Qualitätssicherung und zu der Erkenntnis, dass medizinische Fehlleistungen häufig aus einem Systemversagen resultieren, betreiben Klinken in Deutschland ein Risikomanagement (RM) [1, 17].

Im Fokus des RM stehen Erkennung, Analyse und Vermeidung sog. unerwünschter Ereignisse (UE) [29]. Unter UE werden „Vorkommnisse bzw. Ereignisse [verstanden], die möglicherweise, aber nicht zwangsläufig zu einem konsekutiven Schaden des Patienten führen“ [29]. Ein Bestandteil des RM sind u. a. Critical Incident Reporting Systems (CIRS), welche die Ursachenforschung von UE erleichtern [22]. Zudem stützt der Einsatz von Checklisten durch standardisierte Vorgehensweisen die Prävention von UE [6]. Grundlegend für ein gutes RM ist darüber hinaus eine konstruktive Sicherheitskultur (SK), welche das Ergebnis einer verbesserten Teamarbeit und einer erhöhten Sensibilität für Patientensicherheit innerhalb der Organisation darstellt [7, 31].

Die SK ist Teil der Organisationskultur von Krankenhäusern und beschreibt zugrunde liegende Normen und Verhaltensmuster, die den Umgang der Organisation mit sicherheitsrelevanten Fragen beeinflussen [4]. Alle Organisationsmitglieder reproduzieren die SK durch ihre Handlungen im klinischen Alltag. Insbesondere Führungskräfte sind darüber hinaus für die transparente Kommunikation von Wertvorstellungen zuständig [4].

Eine konstruktive SK zeichnet sich durch regelmäßige Kommunikation, effektive Teamarbeit und kontinuierliche Weiterentwicklung aus [4, 18]. Dabei ist die Zusammenarbeit von Vertrauen und Wertschätzung geprägt [11, 25]: Teambesprechungen finden auch über die eigene Fachdisziplin hinaus statt und haben zum Ziel, gemeinsame Wertvorstellungen zu stabilisieren und das Team für die Patientensicherheit zu sensibilisieren [4]. Organisationsmitglieder erhalten ungeachtet ihrer hierarchischen Position die Möglichkeit, sicherheitsrelevante Fragen anzustoßen [4]. Kommt es zu Fehlhandlungen, können diese in einfach zugängliche Meldesysteme eingetragen werden. Grundlegend hierfür ist ein vertrauensvoller Umgang mit der gemeldeten Information und Strafffreiheit für den Melder [25]. Die Aufarbeitung von UE sollte anschließend problem- und nicht personenzentriert erfolgen [25]. Dies bedeutet, dass Kontext und Bedingungen der Fehlerentstehung in den Fokus gerückt werden, um UE im Sinne einer kontinuierlichen Weiterentwicklung aufzuarbeiten und daraus für die Zukunft zu lernen [25].

Zur Erhebung der SK in Klinken stehen international quantitative Messinstrumente, wie der Safety Attitudes Questionnaire*,* zur Verfügung [11, 23]. Ob und welche Messinstrumente in Deutschland zur Anwendung kommen, ist nach aktuellem Forschungsstand nicht bekannt. Eine Studie zum klinischen RM konnte zeigen, dass 25 % der befragten Krankenhäuser regelmäßig Mitarbeiterbefragungen zur wahrgenommenen SK durchführen [17]. Aussagen zur gegenwärtigen SK in Krankenhäusern können daher vorrangig auf Basis theoretisch-konzeptioneller und teilweise veralteter Betrachtungen getroffen werden [14]. Um zielführende Strategien zur Verbesserung der SK zu entwickeln, ist nicht nur die Charakteristik der SK, sondern darüber hinaus ein tiefer gehendes Verständnis über die zugrunde liegenden Annahmen der Organisationsmitglieder notwendig [16]. Dazu werden seit geraumer Zeit qualitative Untersuchungsmethoden, wie z. B. Interviews, gefordert [11, 16]. Während quantitative Studien mithilfe eines statistisch ausgewerteten Datensatzes auf die Generalisierbarkeit der Ergebnisse abzielen, steht in der qualitativen Forschung die detaillierte Einzelfallanalyse im Fokus. Damit soll ein bestimmtes gesellschaftliches Phänomen beschrieben werden.

Der Fachbereich Orthopädie und Unfallchirurgie (O&U) verzeichnete im Jahr 2018 insgesamt 1690 festgestellte Behandlungsfehler und Risikoaufklärungsmängel und hob sich damit deutlich von anderen Fachdisziplinen ab (z. B. Allgemeinchirurgie 680, innere Medizin 455) [5]. Der Umgang mit UE kann daher in diesem Fachbereich als hochrelevant angesehen werden. Die vorliegende Studie untersuchte mittels qualitativer Forschungsmethoden die ärztliche Perspektive auf die SK in der O&U mit dem Ziel, die relevanten Bestandteile einer SK zu identifizieren und zu untersuchen, wie Ärzte der O&U den Umgang mit UE im klinischen Alltag wahrnehmen.

## Studiendesign und Untersuchungsmethoden

Die vorliegende Studie wurde im Rahmen des Masterstudiengangs „Versorgungsforschung und Implementierungswissenschaft im Gesundheitswesen“ an der Medizinischen Fakultät Heidelberg als Masterarbeit zur Erlangung des akademischen Grads „Master of Science“ durchgeführt.

Der Umgang mit UE in der O&U wurde in 14 Einzelinterviews mit Ärzten der O&U exploriert.

### Rekrutierungsstrategie

Die Auswahl der Studienteilnehmer erfolgte zielgerichtet („purposive sampling“). Diese Methodik stellt ein gängiges Vorgehen in der qualitativen Forschung dar, um einen möglichst hohen Informationsgehalt zu einem unterrepräsentierten Forschungsthema zu generieren [15]. Es wird dabei eine maximale Variation hinsichtlich Geschlecht, Hierarchiezugehörigkeit, Berufsjahren und Arbeitgebern der Studienteilnehmer angestrebt. Ein Arzt aus der O&U fungierte als Gatekeeper und listete 20 potenzielle Studienteilnehmer mit Kontaktdaten. Die Kontaktaufnahme mit diesen Personen erfolgte per E‑Mail. Es erklärten sich 14 Personen nach Erstkontakt zu einem persönlichen oder telefonischen Interview bereit. Nach 10 Tagen wurde an die verbleibenden 6 Personen eine Erinnerung versendet, woraufhin 5 Personen eine Teilnahme aus Zeitmangel absagten. Eine Person meldete sich nicht zurück.

### Beschreibung des Sample

Es wurden 14 Ärzte aus dem Fachbereich O&U rekrutiert, davon 4 Chefärzte, 7 Oberärzte und 3 Assistenzärzte. Die Teilnehmer hatten eine durchschnittliche Berufserfahrung von 16 Jahren (1 bis 37 Jahre). Alle Ärzte waren männlichen Geschlechts und in 8 verschiedenen Krankenhäusern in Deutschland und der Schweiz tätig. Zehn Teilnehmer waren in Krankenhäusern der Maximalversorgung angestellt (Universitätskliniken, berufsgenossenschaftliche Kliniken und Bundeswehrkrankenhäuser). Ein Arzt arbeitete im Krankenhaus der Schwerpunktversorgung und 3 in Krankenhäusern der Grund- und Regelversorgung. Zwei Ärzte berichteten im Interview von ehemaligen Arbeitsstellen, da sie kurz zuvor an einen neuen Arbeitsplatz wechselten.

### Datenerhebung

Vor Beginn der Interviews wurden die Teilnehmer mündlich über datenschutzrechtliche Hinweise und den Inhalt der Studie aufgeklärt, anschließend erfolgte die schriftliche Einwilligung. Persönliche Daten wurden anhand eines Kurzfragebogens erfasst [32].

Die Gespräche fanden von Juli bis November 2019 statt. Vier Interviews fanden am Arbeitsplatz und 10 in der Freizeit der Ärzte statt. Neun Interviews fanden telefonisch und 5 persönlich statt. Die Interviews wurden mithilfe eines halbstrukturierten Leitfadens (Zusatzmaterial online 1) durchgeführt, der ausschließlich offene Fragen enthielt, um die Wahrnehmung der Ärzte möglichst unvoreingenommen erfassen zu können [26, 32]. Alle Interviews wurden von I.H. durchgeführt, tonaufgezeichnet und anschließend wörtlich anhand zuvor festgelegter Transkriptionsregeln transkribiert [30]. Die Interviews dauerten im Mittel 45 min (20–68 min).

### Datenanalyse

Das Datenmaterial wurde zunächst in Anlehnung an die Thematic Analysis (TA) nach Braun und Clarke [2] organisiert. Die TA [2] wird zur induktiven Analyse von Erfahrungen der Studienteilnehmer empfohlen und eignet sich daher für die vorliegende Studie. Hierfür wurde das vorliegende Datenmaterial durch Worte oder Sätze iterativ kodiert [2], (Zusatzmaterial online 2).

Dann wurden die einzelnen Kodierungen anhand einer deduktiven Framework-Analyse in das Yorkshire Contributory Factors Framework (YCFF) [13] eingeordnet. Das YCFF wurde auf Basis eines systematischen Reviews entwickelt und eignet sich zur Identifikation organisationaler Faktoren, welche die SK im klinischen Kontext beeinflussen [13]. Von den insgesamt 20 Faktoren des YCFF wurden 10 zur Datenanalyse verwendet (Zusatzmaterial online 3). Diese wurden anschließend nach Aussagekräftigkeit der zugeordneten Kategorien priorisiert [2]. Zur Organisation der Daten wurde MAXQDA 2018.2 (Verbi Software GmbH, Berlin) verwendet.

Die Interpretation der Themen erfolgte induktiv anhand des vorliegenden Datenmaterials [2]. Dabei wurde nach möglichen Zusammenhängen zwischen den Themen gesucht, um Wechselwirkungen und Einflüsse auf die SK zu identifizieren und beschreiben. Die Ergebnisse wurden in einem Seminar des Studiengangs Versorgungsforschung und Implementierungswissenschaft im Gesundheitswesen der Universität Heidelberg unter der Leitung von C.U. und R.P.D. diskutiert. Zudem wurden die befragten Ärzte um Prüfung der Ergebnisse gebeten („member checking“) [32]. Sieben meldeten sich zurück und bestätigten die dargestellte SK als realitätsnah.

### Ethikvotum

Das positive Ethikvotum wurde am 22.05.2019 bei der Ethikkommission der medizinischen Fakultät der Universität Heidelberg eingeholt (S356-2019).

## Ergebnisse

**Figure 1:**
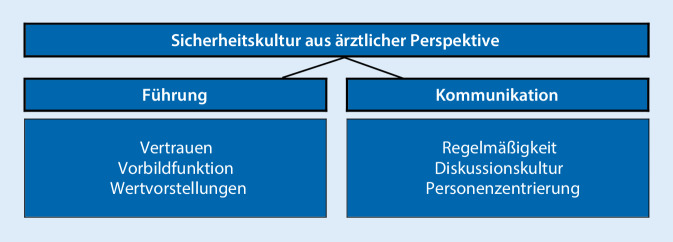

Die Analyse des Datenmaterials ergab, dass die Kategorien „Führung“ und „Kommunikation“ die relevanten Bestandteile der SK in der O&U darstellen (Abb. 1).

## Führung als Bestandteil der Sicherheitskultur

Die Führungskultur zeichnet sich aus Sicht der befragten Ärzte durch einen vertrauensvollen Umgang mit UE, vorbildhaftes Verhalten des Chefarztes sowie gleiche Wertevorstellungen im Team aus.

### Vorleben eines vertrauensvollen Umgangs

Ärzte unterschiedlicher Kliniken beschrieben, von Führungskräften zur Meldung von UE in Meldesystemen wie CIRS aufgefordert zu werden. Alternativ könnten Fehlhandlungen bei zuständigen Ober- oder Chefärzten persönlich gemeldet werden. Laut den Befragten erfordert dies einen vertrauensvollen Umgang im Team. Alle Ärzte formulierten, dass Chefärzte dieses Vertrauen generieren, indem sie eigene Fehlhandlungen zur Diskussion stellen und so v. a. jüngeren Mitarbeitern als Vorbild dienen.

Es ist nicht so, dass die Komplikationen des Chefs nicht gezeigt werden dürfen, sondern gerade die werden gezeigt. Um den anderen auch den Mut zu geben …, dass sie mit Fehlern offen umgehen. – *Chefarzt* [I13]

Sobald … die Jüngeren das Gefühl haben, die Älteren gestehen ihre Komplikationen nicht ein, dann werden sie es auf gar keinen Fall selber tun.* – Oberarzt* [I05]

Telefonische Erreichbarkeit von erfahrenen Ärzten bei Behandlungsentscheidungen wurde von den Assistenzärzten als fördernd für das Vertrauen im Team gesehen. Da das Hierarchiegefälle dieser Kommunikation im Wege stehen könne, bemühen sich die befragten Ober- und Chefärzte laut eigener Aussagen um flache hierarchische Strukturen im Team. Jedoch wurde auch beschrieben, dass dies nicht von allen Kollegen so gelebt wird.

Ich habe … schon versucht, kein hierarchischer Chef zu sein und damit auch das Vertrauen aufzubauen, dass wenn jemand ein Problem hat, einfach bei mir anzurufen. – *Chefarzt* [I04]

Die Hierarchie hat dahingehend Nachteile, dass sich die jungen Kollegen … nicht aus dem Hinterhalt trauen, wenn sie Probleme haben. … Weil [das] von oben her nicht toleriert wird und gesagt wird: „… Ich will da keinen Kontakt haben.“ – *Oberarzt* [I11]

Sanktionen gegen den Melder von UE und emotionale Entgleisungen von Chefärzten wie das Anschreien oder Beschimpfen von Mitarbeitern verhinderten eine offene Kommunikation über UE. Ein Chefarzt erläuterte, dass Angst vor solchen Reaktionen die Meldung von UE hemmt:

Es gibt ja Chefs, die scheißen die Leute gleich zusammen und produzieren damit Angst. Respekt darf man schon produzieren, aber Angst nicht. Angst führt dazu, dass die Leute eben nichts mehr sagen. – *Chefarzt* [I10]

### Werte als Basis der Teambildung

Die befragten Chefärzte beschrieben ihr Bestreben, ein homogenes Team auf Basis gleicher Wertvorstellungen zu bilden. Dies erleichtere die Zusammenarbeit und eine offene Kommunikation über UE. Alle Chefärzte erläuterten, dass Mitarbeiter, die nicht die geforderte SK leben, aus dem Team entfernt werden sollten.

Leider reicht ein schwarzes Schaf in diesem Team … Wenn da einer dabei ist, der … Fehler immer von sich weist, dann macht der das System kaputt. … Es hat lange gedauert, bis man das erkannt hat, aber ich habe es geschafft, dass der gehen musste. – *Chefarzt* [I13]

## Kommunikation über unerwünschte Ereignisse

Von den befragten Ärzten wurden 3 Besprechungstypen benannt, in denen über UE kommuniziert wird: tägliche Fallbesprechungen, Komplikationsbesprechungen und Morbiditäts- und Mortalitätskonferenzen (M&M). Alle Befragten sahen die Verantwortung für Stattfinden und inhaltliche Gestaltung von Besprechungen bei den Chefärzten. Anwesenheit der Führungskräfte bei Besprechungen wurde von den befragten Ärzten erwartet. Die Konferenzen sollten regelmäßig abgehalten werden, um die Diskussion über UE als Routine zu etablieren und einen Lerneffekt für das Team zu generieren.

Ich selber würde einen Fehler viel eher im Zweiergespräch eingestehen können als in einer größeren Gruppe. … Natürlich [ist] der Lernwert … für die Gruppe dann nicht vorhanden. Deswegen finde ich diese Gruppenveranstaltungen schon wichtig. Auch um … zu zeigen, dass Komplikationen zu unserem Geschäft dazugehören. – *Oberarzt* [I05]

### Unterschiedliche Diskussionskultur

Die Diskussionskultur in Besprechungen wurde sehr unterschiedlich beschrieben. Die Befragten erläuterten, dass sich einige Chefärzte um hierarchiefreie Kommunikation bemühen, während andere die beteiligten Operateure in den Fokus rücken und so eine personenzentrierte Perspektive auf UE fördern.

Es soll nicht darum gehen, wer dabei war …, sondern es soll … um das Management dieses Falls gehen. Dass es am Ende dann doch immer so ist, dass der Chef fragt: „Wer war das jetzt eigentlich?“, das ist … natürlich vollkommen unnötig. – *Oberarzt* [I02]

Man kann tatsächlich bei uns … immer sagen: „Darf ich das [Röntgenbild] nochmal sehen? Ist das jetzt wirklich gut?“ … Also das lässt [der Chefarzt] zu und damit ist die Diskussionskultur sehr ausgeprägt*. *– *Oberarzt* [I05]

Zudem wurde die Unterschiedlichkeit thematisierter Fälle in Besprechungen deutlich: Während in manchen Kliniken auch Beinahe-Ereignisse in Besprechungen diskutiert würden, thematisierten andere Kliniken UE nur dann, wenn sie schriftlich dokumentiert waren oder der Patient zu Schaden kam. Es wurde beschrieben, dass in manchen Kliniken UE des Chefarztes nicht gezeigt und ausschließlich UE anderer Häuser zur Diskussion gestellt würden. Die Ärzte berichteten, dass Besprechungen in einigen Kliniken zum Wissensaustausch genutzt würden, während sie in anderen Häusern weniger umfangreich stattfänden.

Wir diskutieren in den Besprechungen schon ein bisschen mehr … Also ich sehe eigentlich diese Besprechungen nicht nur da drin, dass man die Themen abhakt, sondern das ist jedes Mal eine Weiterbildungs- oder Fortbildungsveranstaltung. – *Chefarzt* [I07]

Am Ende der Indikationsbesprechung hat auch keiner mehr groß Lust, lange über Komplikationen zu sprechen, da werden die halt kurz erzählt. – *Oberarzt* [I02]

### Personenzentrierung in Besprechungen

Interdisziplinäre Besprechungen wie die M&M wurden von den Befragten als sinnvoll angesehen, sofern verschiedene Fachdisziplinen an der Entstehung des UE beteiligt waren. Als Vorteile der M&M wurden genannt, dass mehrere Perspektiven zur Aufarbeitung von UE beitragen und ein größeres Publikum aus den Vorfällen lernen könnte. Interdisziplinäre Besprechungen wurden als emotionaler und von Vorurteilen geprägt empfunden als die der eigenen Fachdisziplin.

Manche Anästhesisten haben so ein Minderwertigkeitsgefühl Chirurgen gegenüber. … Die [Anästhesisten] fühlen sich als die Intellektuellen in diesem ganzen Gehabe dort. Und wenn du denen dann auch noch einen Fehler nachweist oder ihn diskutierst, dann schalten die auf stur. – *Chefarzt *[I13]

Weiter wurde erläutert, dass Fälle in M&M oft aus persönlichen Gründen ausgewählt würden, um verantwortliche Operateure in den Fokus zu stellen. Einige Ärzte beschrieben, sich deshalb bestimmte Aussagen anzueignen, um sich vor den Kollegen rechtfertigen zu können.

Am Anfang … habe ich das sehr persönlich genommen, weil ich immer einen hohen Anspruch an mich selbst hatte. Und nach einer gewissen Zeit nimmt man es nicht mehr so persönlich. … Zumindest nach außen legt man sich dann einen Standardsatz zu: „Ich habe das anders gewertet“, oder so. – *Assistenzarzt* [I12]

## Diskussion

Alle befragten Ärzte erwarteten vom Chefarzt, eine konstruktive SK vorzuleben. Dass die Führungskräfte dabei v. a. für jüngere Mitarbeiter ein Vorbild darstellen, wird durch eine Metaanalyse qualitativer Studien aus verschiedenen medizinischen Fachdisziplinen bestätigt [19]. Die befragten Chefärzte wählten ihre Teams auf Basis von Wertvorstellungen aus, was die Wichtigkeit des Handelns nach gemeinsamen Werten zeigt. Zudem ist bereits bekannt, dass das Handeln nach ethischen Grundsätzen v. a. für Führungskräfte eine elementare Kompetenz darstellt [3, 10, 12]. Insbesondere Chefärzte sind für die Vorgabe eines ethischen Rahmens zuständig, in dem Ärzte weitestgehend autonom handeln können [10]. Bei wiederholter Nichtbefolgung dieser impliziten Vorgaben erläuterten die befragten Chefärzte, dass entsprechende Ärzte aus dem Team zu entfernen seien. Ähnliche Ergebnisse konnten auch in einer ethnografischen Studie über intensivmedizinisches Personal gezeigt werden [28].

Dass Chefärzte nicht nur einen Handlungsrahmen vorgeben, sondern durch ihr Verhalten die Handlungen des ärztlichen Teams beeinflussen, ist aus anderen Fachdisziplinen bekannt [3, 21, 27]. Es gibt konkrete Hinweise darauf, dass eine straffreie Meldung von UE und die Möglichkeit des Lernens aus Fehlern die Frequenz von Fehlermeldungen beeinflusst [21]. Die Ergebnisse dieser Studie zeigen darüber hinaus, dass für die Meldung von UE ein vertrauensvoller Umgang im Team grundlegend ist und die Chefärzte dieses durch das Eingestehen eigener Fehlhandlungen vorleben. Dagegen konnten Sanktionen gegen den Melder eines Fehlers und emotionale Reaktionen des Chefarztes wie auch bei Morrow et al. [19] als eine Barriere für die Kommunikation von UE identifiziert werden.

Ein vertrauensvoller Umgang mit dem Melder als Bestandteil einer konstruktiven SK [25] wurde zwar von vielen Befragten als erstrebenswert beschrieben, aber nicht von allen so erlebt. Vor allem die interdisziplinäre M&M stand aufgrund einer häufig wahrgenommenen Personenzentrierung in der Kritik. Die vorliegenden Ergebnisse zeigen, dass das Fokussieren auf vermeintlich schuldige Ärzte ein Verschweigen von eigenen Fehlhandlungen fördert, um vor den Kollegen nicht bloßgestellt zu werden. Dies wird durch eine weitere Studie [19] bekräftigt. Hinsichtlich der interdisziplinären Kommunikation wurde deutlich, dass diese von Stereotypen begleitet ist. So wurden Anästhesisten beschuldigt, die Aufarbeitung von UE durch das Negieren von Fehlhandlungen zu behindern. Gillespie et al. [8] fanden hinsichtlich der Teamarbeit bei Operationen heraus, dass die Zusammensetzung des Teams sowohl die Kommunikation als auch den Inhalt des Informationsaustausches beeinflusst. Der Grund hierfür könnte in der beruflichen Identifikation von Chirurgen liegen, die durch einen hohen Individualismus geprägt ist und die Teamarbeit einschränken kann [8].

Streng hierarchische Strukturen konnten ebenfalls als Hindernis für die Kommunikation von UE identifiziert werden. Dies zeigt sich konsistent mit Ergebnissen weiterer Studien [9, 20, 24]. Schwappach et al. [24] untersuchten die Kommunikation über sicherheitsrelevante Themen in deutschsprachigen Krankenhäusern und konnten zeigen, dass 20 % der Befragten sicherheitsgefährdende Handlungen zwar beobachteten, aber aus Gründen der sozialen Konstellation nicht meldeten.

In dieser Studie beschrieben Ärzte, UE in 3 verschiedenen Besprechungstypen aufzuarbeiten, was den Anforderungen einer konstruktiven SK entspricht [4]. Dabei wurde die Notwendigkeit der Regelmäßigkeit von Teamsitzungen betont, um sie als Rahmenbedingungen der Kommunikation zu etablieren. Dies konnte auch in einer Fokusgruppenstudie in dänischen Universitätskliniken gezeigt werden [20]. Auffällig war in der vorliegenden Studie die Varianz der inhaltlichen Gestaltung zwischen den verschiedenen Krankenhäusern. Die Ergebnisse zeigen, dass durch die fehlende Standardisierung oft nicht das Lernen aus Fehlern im Mittelpunkt steht, sondern eine personenzentrierte Fallauswahl ermöglicht wird.

### Stärken und Limitationen

In der vorliegenden Studie wurde die SK anhand der TA nach Braun und Clarke [2] und mithilfe einer ergänzenden Framework-Analyse exploriert. So konnten relevante Bestandteile der SK aus ärztlicher Perspektive anhand eines dichten Datenmaterials erläutert werden. Die Befragung von Ärzten aus 8 verschiedenen Kliniken mit unterschiedlicher Hierarchiezugehörigkeit und Berufserfahrung ermöglicht einen fundierten Einblick in ein bisher unterrepräsentiertes Forschungsgebiet. Die Interpretation der Studienergebnisse wurde durch die befragten Ärzte selbst als realitätsnah bestätigt [26, 30].

Durch das Heranziehen eines Gatekeeper erfolgte die Rekrutierung von Studienteilnehmern zielgerichtet und zeitnah. Im Sample könnten sich jedoch hauptsächlich Ärzte befunden haben, die einer Studienteilnahme aufgeschlossen und für eine konstruktive SK sensibilisiert waren. Zudem beruhte der Umfang des Sample auf Aspekten der Machbarkeit und nicht ausschließlich auf inhaltlicher Sättigung der erhobenen Daten, was die Validität der Ergebnisse beschränken kann. Da ausschließlich männliche Ärzte befragt wurden, konnten geschlechterbezogene Perspektiven auf die SK nicht adressiert werden. Die Erhöhung der Beteiligung von Ärzten aller Geschlechter könnte ein Ziel weiterführender Forschung sein, um das Thema noch umfangreicher beleuchten zu können.

## Fazit für die Praxis

- Mitarbeiterbefragungen zur wahrgenommenen Sicherheitskultur (SK) finden in deutschen Kliniken nicht flächendeckend statt. Zudem ist unbekannt, welche Messinstrumente hierfür verwendet werden.
- Zukünftige Forschung zur SK in der Orthopädie und Unfallchirurgie (O&U) sollte sowohl die Bedeutsamkeit hierarchischer Strukturen als auch geschlechterspezifische Faktoren im Umgang mit unerwünschten Ereignissen (UE) und der chefärztlichen Rolle genauer untersuchen.
- Das Einbeziehen von Führungskräften in die Entwicklung von Strategien zur Verbesserung der SK erscheint unabdingbar: Führungskräfte sollten sich stets bewusst sein, dass sie aufgrund ihrer Vorbildfunktion eine zentrale Rolle in der Beeinflussung der SK einnehmen.
- Die straffreie Meldung von UE, das Eingestehen eigener Fehlhandlungen durch die Führungskraft sowie Vertrauen im Team sind grundlegend für die Kommunikation über UE.

## Caption Electronic Supplementary Material






