# Supplementary material for: Safety culture in orthopedics and trauma surgery: A qualitative study of the physicians’ perspective
Source: Unfallchirurg. 2020 Nov 10;124(6):481–8. [Article in German] doi: 10.1007/s00113-020-00917-0 (PMC8159809; doi:10.1007/s00113-020-00917-0)
Supplement: Supplementary file 1 [file 113_2020_917_MOESM1_ESM.pdf]

Zusatzmaterial 1: Interview-Leitfaden

I. Einführung

F1 Wie haben Sie sich für den Fachbereich der Unfallchirurgie entschieden?

F2 Beschreiben Sie mir doch bitte einmal den Alltag in der Klinik.

II. Umgang mit unerwünschten Ereignissen (UE) im Team

Fallbeispiel: Ein Patient kommt 2 Wochen nach Hüftprothesen-OP in die Ambulanz mit Schwellung, Rötung, Schmerz im operierten Gebiet. Der behandelnde Arzt spricht mit dem Patienten und untersucht ihn. Es wird jedoch keine Blutentnahme oder Punktion durchgeführt. Der Patient wird nach Hause geschickt und kommt ein paar Tage später mit deutlich stärkeren Symptomen wieder.

F1 Wie handelt der behandelnde Arzt (handeln Ihre Kollegen) nach Ihrer Erfahrung üblicherweise in dieser Situation?

F2 Fallen Ihnen ähnliche Fallbeispiele (Kontrastbeispiele) ein, die dem genannten ähneln?

F3 Welche unterschiedlichen Umgangsweisen mit solchen Fällen können Sie innerhalb Ihres Kollegiums beobachten?

III. Einflussfaktoren auf den Umgang mit UE

F1 Was beeinflusst denn den Umgang mit UE?

F2 Wenn Sie entscheiden dürften: Welche Änderungen würden Sie vornehmen, damit noch offener über UE gesprochen wird?

IV. Eigener Umgang mit UE

F1 Rückblickend betrachtet: Wie hat sich Ihr eigener Umgang mit UE während der beruflichen Laufbahn verändert?

V. Abschluss

F1 Haben wir einen Aspekt noch nicht angesprochen, den Sie gerne thematisieren möchten?
