# Supplementary material for: Safety culture in orthopedics and trauma surgery: A qualitative study of the physicians’ perspective
Source: Unfallchirurg. 2020 Nov 10;124(6):481–8. [Article in German] doi: 10.1007/s00113-020-00917-0 (PMC8159809; doi:10.1007/s00113-020-00917-0)
Supplement: Supplementary file 2 [file 113_2020_917_MOESM2_ESM.pdf]

Zusatzmaterial 2: Beispiele für induktive Kodierungen

Kodierungsbeispiel für „Vorbildfunktion“:

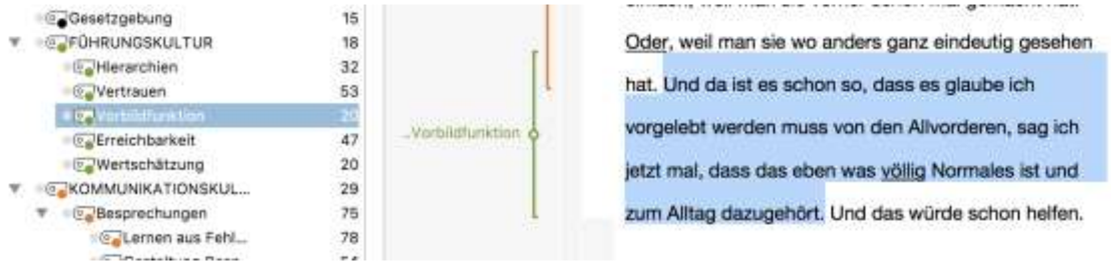

Kodierungsbeispiel für „Personenzentrierung“:

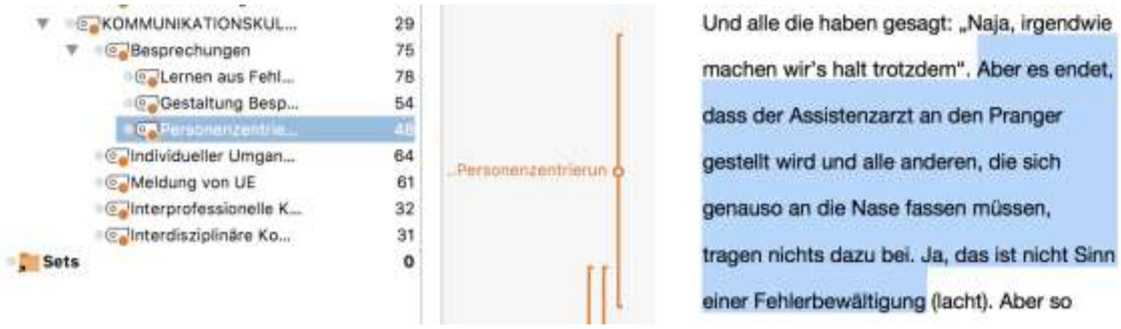

(Abbildungen aus MAXQDA)
