# Supplementary material for: Safety culture in orthopedics and trauma surgery: A qualitative study of the physicians’ perspective
Source: Unfallchirurg. 2020 Nov 10;124(6):481–8. [Article in German] doi: 10.1007/s00113-020-00917-0 (PMC8159809; doi:10.1007/s00113-020-00917-0)
Supplement: Supplementary file 3 [file 113_2020_917_MOESM3_ESM.pdf]

Zusatzmaterial 3: Verwendete Faktoren des *Yorkshire Contributory Factors Framework* [1]

Faktor	Definition
Teamfaktoren	Veränderbare Faktoren, die im Zusammenhang mit interprofessionellem Zusammenarbeiten stehen
Individuelle Faktoren	Merkmale der Person, die die Versorgung erbringt: Persönlicher Charakter, Unerfahrenheit, Stress
Verantwortlichkeit	Vorhandensein klarer Verantwortlichkeiten unter den Mitarbeitern
Auslastung des Personals	Aktivität und Druck während einer Arbeitsschicht
Führung & Leitung	Verfügbarkeit und Qualität der Führung und Leitung
Ausbildung & Weiterbildung	Zugang zu organisations- und aufgabenbezogenen Seminaren
Richtlinien & Vorschriften	Vorhandensein, Qualität und Aussagekraft formaler und niedergeschriebener Richtlinien zur Anleitung von Tätigkeiten
Außenpolitischer Kontext	(Nationale) Richtlinien, die sich auf die Ressourcen einer Klink auswirken
Kommunikationssysteme	Effektivität des Informationsaustauschs zw. Versorgern, Patienten und Abteilungen (Dokumentation, Besprechungen, Übergaben)
Sicherheitskultur	Werte einer Organisation, Umgang und Management von UE, Lernen aus UE

1. Lawton R, Mceachan RR, Giles SJ et al. (2012) Development of an evidence-based framework of factors contributing to patient safety incidents in hospital settings: a systematic review. BMJ Qual Saf 21:369-380
